# Value of thyroid cancer history in the prognosis of pancreatic cancer: a SEER population-based study

**DOI:** 10.1038/s41598-023-32635-z

**Published:** 2023-04-08

**Authors:** Jun He, Yu Wang, Xiangmei Chen, Wenxiang Chen, Jianyin Zhou

**Affiliations:** Department of Hepatobiliary Surgery, Zhongshan Hospital of Xiamen University, School of Medicine, Xiamen University, Xiamen, China

**Keywords:** Cancer, Oncology

## Abstract

Thyroid cancer patients have a good prognosis, and their long survival increases the likelihood of developing a second primary tumor. Meanwhile, pancreatic cancer (PC) has a poor prognosis and therapeutic efficacy. However, the association between prior thyroid cancer and the subsequent PC prognosis is unknown. Herein, we selected pathologically diagnosed PC patients older than 17 between 2010 and 2015 from the SEER database. We used propensity score matching (PSM) to reduce confounding factors between groups and matched each PC patient with a history of thyroid cancer with 10 PC patients without a history of thyroid cancer. Finally, we selected 103 PC patients with prior thyroid cancer and 1030 PC patients without prior thyroid cancer. Then, we analyzed the factors influencing the overall survival (OS) and the cancer-specific survival (CSS) of PC patients. The median overall survival of PC patients with and without a history of thyroid cancer was 12 and 9 months, respectively. The history of thyroid cancer in PC patients reduced the PC-specific mortality (*p* < 0.05). Prior thyroid cancer might be a favorable prognostic factor for PC-specific mortality in PC patients.

## Introduction

Pancreatic cancer (PC) has high malignancy and poor prognosis. Most PC patients develop symptoms at an advanced stage, and very few survive more than five years^1,2^. Advanced PC patients are deprived of surgery, and the main treatment is chemotherapy. With 495,773 new cases in 2020, PC was the seventh leading cause of cancer-related death worldwide and is projected to surpass breast cancer as the second leading cause of cancer-related death in the United States by 2030^3,4^. PC tends to occur in the elderly, with a higher incidence in males than females and a larger gap in countries with higher development indexes^5^.

The number of cancer survivors has greatly increased in recent decades due to advances in early diagnosis and the diversification of anti-cancer drugs^6^. Cancer survivors have a higher risk of developing or dying from secondary primary tumors than the general population^7^. Moreover, thyroid cancer is a tumor with a very good prognosis. Its incidence ranks ninth among malignant tumors and is significantly higher in females than males^4^. However, the long-term survival of thyroid cancer patients increases the likelihood of developing a second primary tumor. For example, hypothyroidism is associated with autoimmune pancreatitis, a potential risk factor for PC^8,9^. Prior thyroid cancer has been found in many studies as a favorable prognostic factor for second primary tumors^10,11^. Thyroid cancer is also beneficial for the survival of subsequent liver cancer^10^ and breast cancer^11^. Nevertheless, research about the association between prior thyroid cancer and PC is rare, and further studies are required on these two cancers with very different prognoses.

Therefore, in the present study, we used SEER*Stat software version 8.4.0 to download datasets from the Surveillance, Epidemiology, and End Results Program (SEER) database. Then, we investigated prognostic factors associated with PC survival and analyzed the association between prior thyroid cancer and PC-specific mortality in PC patients.

## Materials and methods

### Data sources

We retrieved data on PC patients with at least 18 years between 2010 and 2015 from the SEER database. The SEER database provides free access to cancer patient information from over a dozen registries in the United States collected for decades, including demographic information, tumor type, disease stage, treatment strategy, and survival prognosis^12^. We downloaded data from Incidence-SEER Research Plus Data from 18 Registries based on the November 2020 submission. PC was identified under the International Classification of Diseases of Oncology, Third Edition ICD-O-3: C25.0, C25.1, C25.2, C25.3, C25.4, C25.7, C25.8, and C25.9.

### Inclusion and exclusion criteria

**Figure 1:**
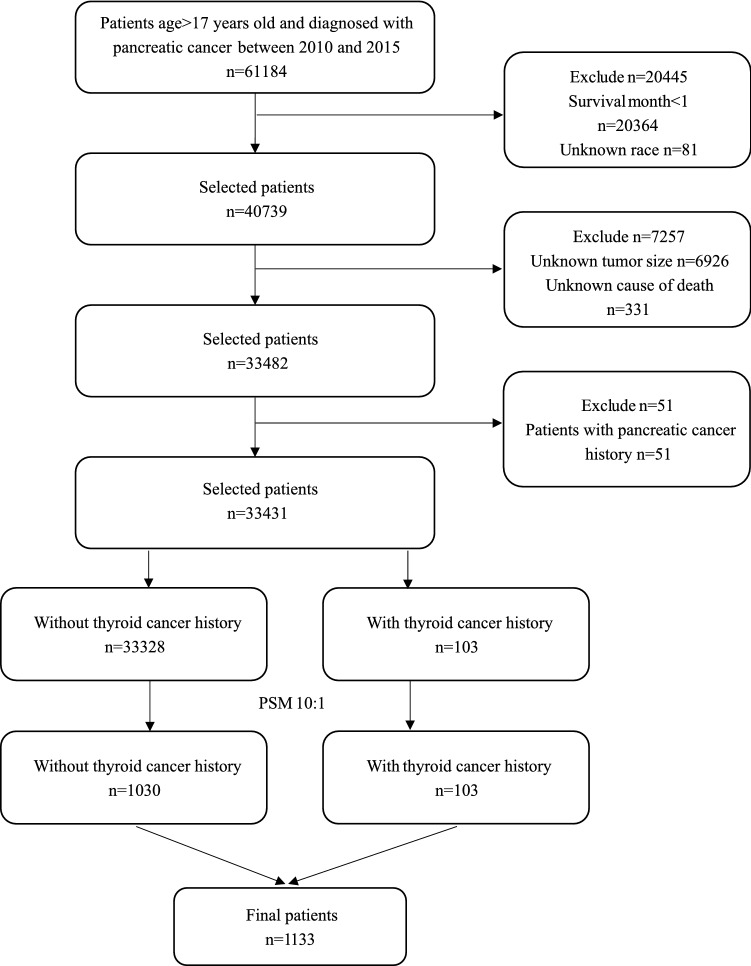
Flowchart of patients’ selection.* PSM* propensity score matching.

The inclusion criteria included: (1) PC patients diagnosed between 2010 and 2015; (2) > 17 years; (3) histologic type code: 8140, 8480, 8500, 8246, 8010, 8012, 8013, 8020, 8021, 8041, 8046, 8070, 8150, 8240, 8244, 8249, 8481, 8490, and 8560; (4) complete survival information. The exclusion criteria were: (1) survival time < 1 month; (2) unknown race; (3) unknown tumor size; (4) unknown SEER cause-specific death classification; (5) patients with a history of PC. The flowchart of patient selection is shown in Fig. 1.

### Patient’s clinical characteristics

We collected patient clinical characteristics, including age at diagnosis, sex, race, the primary site of the tumor, tumor size, grade, histological subtype, AJCC TNM (7th) status, radiotherapy, chemotherapy, surgery, survival time, survival status, and the SEER cause-specific death classification.

### Definition of variables

**Figure 2:**
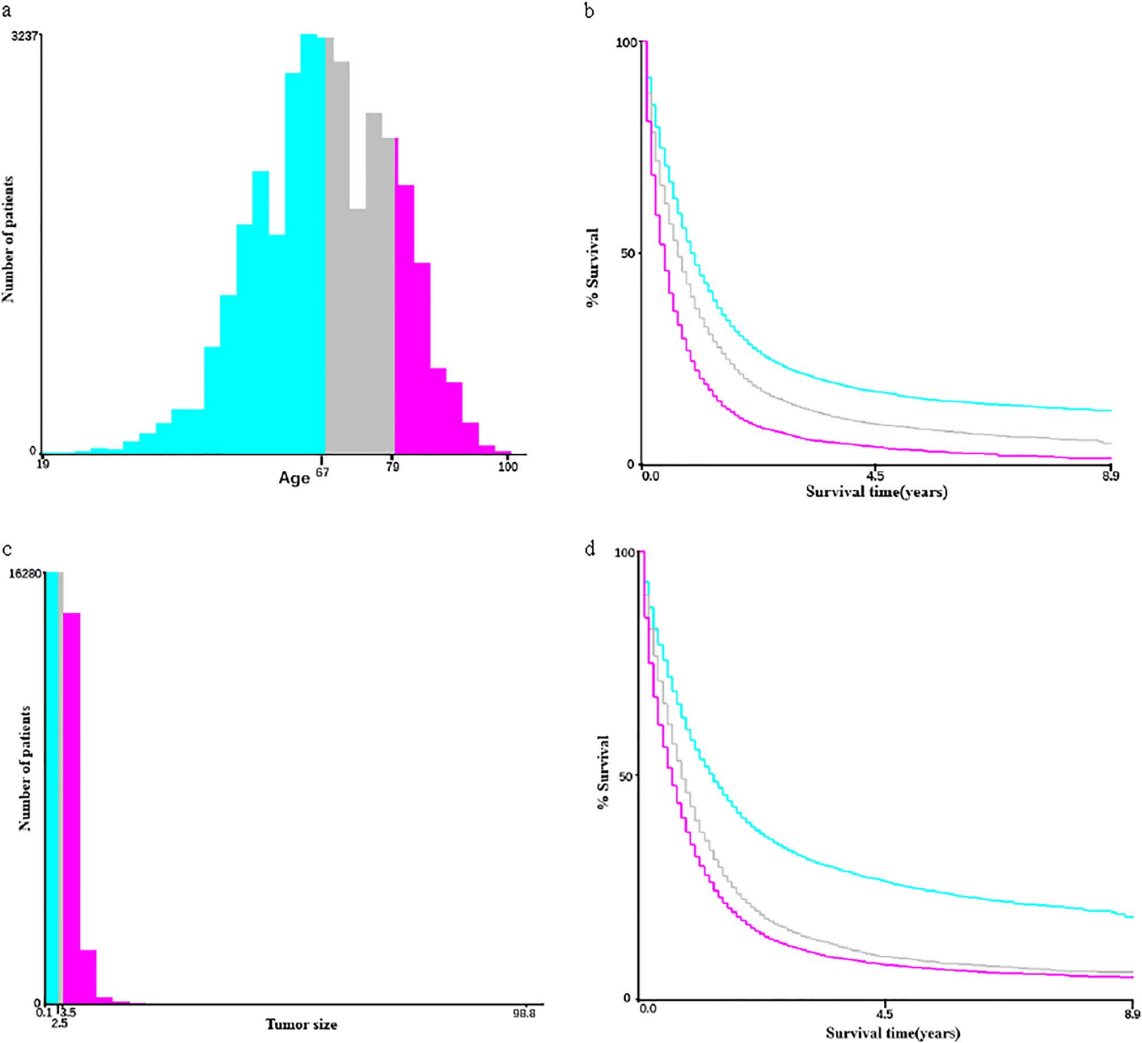
Patient numbers and survival curves generated by X-tile software. (**a**) Number of patients at different ages of diagnosis. (**b**) Survival curves for three age ranges. (**c**) Number of patients with different tumor sizes. (**d**) Survival curves for three tumor size ranges.

Prior thyroid cancer refers to thyroid cancer diagnosed earlier than PC. The identification of thyroid cancer was based on the International Classification of Diseases for Oncology, Third Edition ICD-O-3:C73.9, the thyroid gland. We classified pancreatic cancer into four histological subtypes based on the histological subtype coding: adenocarcinoma, infiltrating duct carcinoma, neuroendocrine carcinoma, and others^13^. We divided the survival status of PC patients into (1) alive; (2) PC-specific death; (3) death due to other causes. The continuous variables (age and tumor size) were divided into three categorical variables based on the optimal cut-off values using X-tile (v. 3.6.1) (Fig. 2). X-tile is a bioinformatics tool that can classify continuous variables into categorical variables based on outcome-based cut-point optimization^14^.

### Statistical analysis

Continuous variables with non-normal distribution are expressed as medians and quartiles [M (Q1, Q3)]. Categorical variables are expressed as numbers and component ratios (n, %). The Mann–Whitney U rank-sum test was used to compare differences among continuous variables with non-normal distribution, while the χ^2^ and Fisher's exact tests were used to compare differences among categorical variables.

The propensity score matching (PSM) can reduce confounding factors and increase the comparability between groups^15^. We used the “Matchlt” R package to implement PSM by taking age and gender as scoring factors. We set the caliper value to 0.1 and the matching ratio to 1:10; that is, each PC patient with a history of thyroid cancer was matched with 10 PC patients without a history of thyroid cancer.

The end events of interest were overall survival (OS) and cancer-specific survival (CSS). OS refers to the time interval of PC patients from diagnosis to death, while CSS refers to the time interval of PC patients from diagnosis to death due to PC. The Kaplan–Meier (KM) method and log-rank test were used for survival analysis, and the OS rate was plotted by the KM method. Univariate and multivariate survival analyses were used to study the association between OS and each variable. In the competing risk model, death due to other causes was considered the competing event. Univariate and multivariate analyses of CSS were also conducted to study the association between PC-specific death and each variable. The competing risk model analysis was implemented using the “Cmprsk” R package.

In the subgroup analysis, PC patients were divided into three subgroups based on histological subtypes: 1. adenocarcinoma and infiltrating ductal carcinoma; 2. neuroendocrine carcinoma; 3. other histological subtypes.

R 4.1.3 and SPSS (v. 26.0, IBM, Armonk, NY, USA) were used for all data analyses. All statistical tests were bilateral, and the cut-off *p*-value for the statistical difference was 0.05.

### Ethics declarations

All data in this paper can be obtained from the SEER database. Since the SEER database is publicly available and anonymous to all patient information, informed consent and ethical approval were not required. This study strictly followed relevant guidelines and regulations to implement all methods.

## Results

### Patients’ clinicopathological baseline information

We selected 33,431 PC patients, 33,328 without and 103 with prior thyroid cancer. Among demographic factors, race and age at diagnosis did not differ between the two groups. Most PC patients were white and younger than 68 years. Meanwhile, the two groups significantly differed in sex, tumor size, the primary site of the tumor, and survival months. PC patients with a history of thyroid cancer had longer survival than those without (*p* < 0.05). Compared to PC patients without a history of thyroid cancer, PC patients with a history of thyroid cancer had a higher rate of women, tumors larger than 3.5 cm or smaller than 2.6 cm, and primary site in the tail of the pancreas (Supplementary Table 1). Most of the 103 thyroid cancer patients had thyroid tumors smaller than 3 cm and underwent surgery. (Supplementary Table 2).

### Patient clinicopathological baseline information after PSM

Based on the PSM, we selected 1133 from 33,328 PC patients, 1030 without and 103 with prior thyroid cancer. Among demographic factors, the two groups did not differ regarding age at diagnosis, sex, and race. Meanwhile, tumor size, radiation therapy, and the primary site of the tumor were significantly different. Compared to PC patients without a history of thyroid cancer, those with a history of thyroid cancer had a higher rate of tumors larger than 3.5 cm or smaller than 2.6 cm, primary site in the tail of the pancreas, and radiation treatment. The median survival of PC patients with and without prior thyroid cancer were 9 and 11 months, respectively, but did not statistically differ (Table 1).

**Table 1:** Patients’ clinicopathological baseline information.

Characteristics	Total (n = 1133)	Without prior thyroid cancer (n = 1030)	With prior thyroid cancer (n = 103)	Statistics	P value
Age at diagnosis, n (%)					
< 68	550 (48.5)	500 (48.5)	50 (48.5)	χ^2^ < 0.001	1
68–79	352 (31.1)	320 (31.1)	32 (31.1)		
> 79	231 (20.4)	210 (20.4)	21 (20.4)		
Sex, n (%)					
Female	792 (69.9)	720 (69.9)	72 (69.9)	χ^2^ < 0.001	1
Male	341 (30.1)	310 (30.1)	31 (30.1)		
Race, n (%)					
Black	139 (12.3)	129 (12.5)	10 (9.7)	χ^2^ = 2.432	0.296
White	793 (70.0)	714 (69.3)	79 (76.7)		
Others	201 (17.7)	187 (18.2)	14 (13.6)		
Tumor size, n (%)					
< 2.6	287 (25.3)	257 (25.0)	30 (29.1)	χ^2^ = 9.943	0.007
2.6–3.5	315 (27.8)	300 (29.1)	15 (14.6)		
> 3.5	531 (46.9)	473 (45.9)	58 (56.3)		
Grade, n (%)					
Well differentiated	133 (11.7)	119 (11.6)	14 (13.6)	Fisher	0.815
Moderately differentiated	209 (18.4)	192 (18.6)	17 (16.5)		
Poorly differentiated	147 (13.0)	131 (12.7)	16 (15.5)		
Undifferentiated	7 (0.6)	7 (0.7)	0 (0)		
Unknown	637 (56.2)	581 (56.4)	56 (54.4)		
Radiotherapy, n (%)					
None/Unknown	965 (85.2)	885 (85.9)	80 (77.7)	χ^2^ = 5.049	0.025
Yes	168 (14.8)	145 (14.1)	23 (22.3)		
Chemotherapy, n (%)					
None/Unknown	496 (43.8)	454 (44.1)	42 (40.8)	χ^2^ = 0.415	0.520
Yes	637 (56.2)	576 (55.9)	61 (59.2)		
Surgery, n (%)					
None/Unknown	798 (70.4)	730 (70.9)	68 (66.0)	χ^2^ = 1.060	0.303
Yes	335 (29.6)	300 (29.1)	35 (34.0)		
AJCC T status, n (%)					
T1	104 (9.2)	95 (9.2)	9 (8.7)	χ^2^ = 2.482	0.648
T2	287 (25.3)	259 (25.1)	28 (27.2)		
T3	456 (40.3)	410 (39.8)	46 (44.7)		
T4	228 (20.1)	213 (20.7)	15 (14.6)		
Unknown	58 (5.1)	53 (5.1)	5 (4.9)		
AJCC N status, n (%)					
N0	634 (56.0)	570 (55.3)	64 (62.1)	χ^2^ = 1.864	0.394
N1	439 (38.7)	404 (39.2)	35 (34.0)		
Unknown	60 (5.3)	56 (5.4)	4 (3.9)		
AJCC M status, n (%)					
M0	675 (59.6)	611 (59.3)	64 (62.1)	χ^2^ = 0.308	0.579
M1	458 (40.4)	419 (40.7)	39 (37.9)		
Histological subtype, n (%)					
Adenocarcinoma	765 (67.5)	697 (67.7)	68 (66.0)	χ^2^ = 1.216	0.754
Infiltrating duct carcinoma	137 (12.1)	123 (11.9)	14 (13.6)		
Neuroendocrine carcinoma	89 (7.9)	83 (8.1)	6 (5.8)		
Other	142 (12.5)	127 (12.3)	15 (14.6)		
SEER cause death classification, n (%)					
Alive	162 (14.3)	145 (14.1)	17 (16.5)	χ^2^ = 4.248	0.120
Death due to other causes	64 (5.6)	54 (5.2)	10 (9.7)		
Death due to pancreatic cancer	907 (80.1)	831 (80.7)	76 (73.8)		
Survival month, M (Q1, Q3)	9.0 (3.0,23.0)	9.0 (3.0,22.0)	11.0 (5.0,29.0)	Z = − 1.293	0.196
Vital status, n (%)					
Alive	162 (14.3)	145 (14.1)	17 (16.5)	χ^2^ = 0.450	0.502
Dead	971 (85.7)	885 (85.9)	86 (83.5)		
Primary site, n (%)					
Head of pancreas	605 (53.4)	549 (53.3)	56 (54.4)	Fisher	0.001
Body of pancreas	186 (16.4)	180 (17.5)	6 (5.8)		
Tail of pancreas	150 (13.2)	126 (12.2)	24 (23.3)		
Pancreatic duct	6 (0.5)	5 (0.5)	1 (1.0)		
Other specified parts of pancreas	31 (2.7)	31 (3.0)	0		
Overlapping lesion of pancreas	95 (8.4)	86 (8.3)	9 (8.7)		
Pancreas, NOS	60 (5.3)	53 (5.1)	7 (6.8)		

*AJCC* American Joint Commission on Cancer(7th), *SEER* the surveillance, epidemiology, and end results.

### Univariate and multivariate Cox survival analyses of OS in PC patients

The median OS of PC patients with and without prior thyroid cancer was 12 and 9 months, respectively, but did not statistically differ (*p* > 0.05) (Supplementary Fig. 1). The KM survival analyses of different subgroups is shown in Supplementary Table 3. The factors that were statistically significant with OS in PC patients in the univariate analysis were age, race, tumor size, grade, the primary site of the tumor, histological subtype, AJCC T status, AJCC N status, AJCC M status, radiotherapy, and surgery. In the multivariate analysis of OS, the age, race, tumor size, grade, histological subtype, AJCC T stage, AJCC N stage, AJCC M stage, chemotherapy, and surgery were statistically significant. The favorable factors for OS included race as other, primary site in the tail of the pancreas, neuroendocrine carcinoma or other histological subtypes, chemotherapy, and surgery. The unfavorable factors for OS included age > 68, tumor > 3.5, moderately differentiated, poorly differentiated, T3, N1, and M1(Table 2). The univariate and multivariate Cox survival analyses in different subgroups of PC patients are presented in Supplementary Table 4.

**Table 2:** Univariate and multivariate Cox survival analyes of OS in PC patients.

Characteristics	Univariate analysis		Multivariate analysis	
	HR (95% CI)	P value	HR (95% CI)	P value
Age (Reference: < 68)				
68–79	1.637 (1.415–1.894)	< 0.001	1.458 (1.253–1.697)	< 0.001
> 79	2.620 (2.222–3.089)	< 0.001	1.917 (1.602–2.295)	< 0.001
Sex (Reference: Female)				
Male	1.059 (0.924–1.215)	0.408	1.125 (0.977–1.295)	0.104
Race (Reference: Black)				
White	0.794 (0.655–0.963)	0.019	0.780 (0.639–0.954)	0.153
Other	0.814 (0.645–1.028)	0.083	0.768 (0.605–0.975)	0.030
Tumor size (Reference: < 2.6)				
2.6–3.5	2.041 (1.700–2.450)	< 0.001	1.213 (0.989–1.487)	0.063
> 3.5	2.215 (1.874–2.617)	< 0.001	1.372 (1.129–1.668)	0.001
Grade (Reference: Well differentiated)				
Moderately differentiated	2.842 (2.103–3.842)	< 0.001	1.958 (1.427–2.687)	< 0.001
Poorly differentiated	4.278 (3.132–5.844)	< 0.001	2.472 (1.780–3.434)	< 0.001
Undifferentiated	3.671 (1.582–8.518)	0.002	1.969 (0.825–4.699)	0.127
Unknown	6.081 (4.615–8.013)	< 0.001	1.87 5(1.380–2.549)	< 0.001
Radiotherapy (Reference: None/Unknown)				
Yes	0.797 (0.670–0.948)	0.010	0.991 (0.820–1.198)	0.924
Chemotherapy (Reference: None/Unknown)				
Yes	0.960 (0.843–1.093)	0.536	0.480 (0.429–0.582)	< 0.001
Surgery (Reference: None/Unknown)				
Yes	0.256 (0.218–0.300)	< 0.001	0.340 (0.268–0.431)	< 0.001
AJCC T status (Reference: T1)				
T2	3.890 (2.811–5.385)	< 0.001	1.394 (0.963–2.018)	0.079
T3	3.845 (2.802–5.277)	< 0.001	1.449 (1.000–2.100)	0.049
T4	4.907 (3.523–6.835)	< 0.001	1.225 (0.823–1.823)	0.318
Unknown	8.497 (5.693–12.683)	< 0.001	1.996 (1.282–3.106)	0.002
AJCC N status (Reference: N0)				
N1	1.124 (0.985–1.281)	0.082	1.192 (1.029–1.382)	0.019
Unknown	2.834 (2.155–3.727)	< 0.001	1.748 (1.315–2.323)	< 0.001
AJCC M status (Reference: M0)				
M1	2.401 (2.110–2.732)	< 0.001	1.826 (1.557–2.142)	< 0.001
Primary site (Reference: Head of pancreas)				
Body of pancreas	0.911 (0.762–1.089)	0.307	0.844 (0.698–1.021)	0.081
Tail of pancreas	0.744 (0.607–0.913)	0.005	0.789 (0.630–0.987)	0.038
Pancreatic duct	1.518 (0.679–3.394)	0.310	1.270 (0.562–2.872)	0.566
Other specified parts of pancreas	0.860 (0.576–1.284)	0.461	0.840 (0.559–1.262)	0.401
Overlapping lesion of pancreas	1.081 (0.859–1.361)	0.506	1.007 (0.791–1.283)	0.953
Pancreas, NOS	1.236 (0.927–1.648)	0.148	1.199 (0.887–1.620)	0.237
Histological subtype (Reference: Adenocarcinoma)				
Infiltrating duct carcinoma	0.510 (0.418–0.622)	< 0.001	1.0245 (0.815–1.288)	0.836
Neuroendocrine carcinoma	0.124 (0.086–0.180)	< 0.001	0.153 (0.103–0.227)	< 0.001
Other	0.436 (0.351–0.543)	< 0.001	0.552 (0.430–0.708)	< 0.001
Prior thyroid cancer history (Reference: without)				
With	0.876 (0.702–1.093)	0.240	0.824 (0.652–1.041)	0.104

*OS* overall survival, *AJCC* American Joint Commission on Cancer(7th), *SEER* the surveillance, epidemiology, and end results, *HR* hazard ratio, *CI* confidence interval.

### Association between the history of thyroid cancer and PC-specific mortality in PC patients

**Figure 3:**
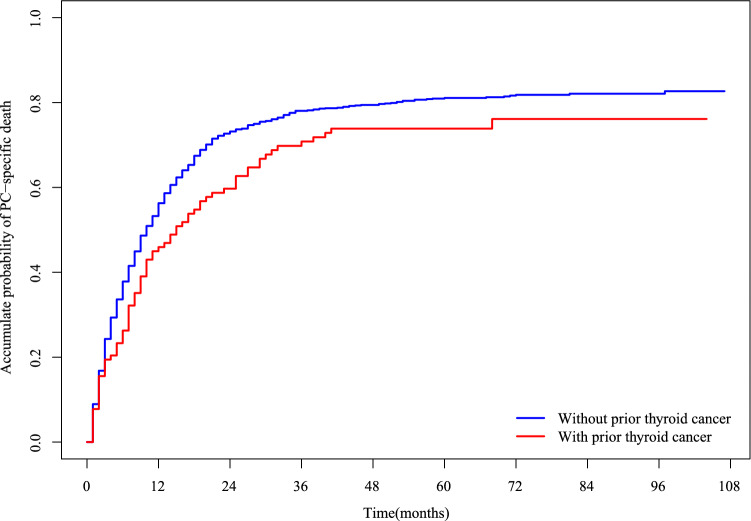
Cumulative incidence of PC-specific death in PC patients with and without prior thyroid cancer.* PC* pancreatic cancer.

The cumulative PC-specific mortality in PC patients with and without a history of thyroid cancer is presented in Fig. 3. PC patients with a history of thyroid cancer had lower PC-specific mortality than those without (*p* < 0.05). In the Fine and Gray's competing risk model, the multivariate analysis of CSS in PC patients is shown in Table 3. The history of thyroid cancer was associated with a 0.278-fold reduction in PC-specific mortality [Hazard Ratio (HR) = 0.722, 95% confidence interval (CI): 0.542–0.961, *p* = 0.026]. Therefore, prior thyroid cancer might be a favorable prognostic factor for PC-specific mortality in PC patients.

**Table 3:** Multivariate analysis of CSS in PC patients.

Characteristics	HR (95% CI)	P value
Age (Reference: < 68)		
68–79	1.259 (1.075–1.474)	0.004
> 79	1.460 (1.192–1.789)	< 0.001
Sex (Reference: Female)		
Male	1.017 (0.872–1.186)	0.830
Race (Reference: Black)		
White	0.756 (0.618–0.925)	0.007
Other	0.805 (0.627–1.034)	0.090
Tumor size (Reference: < 2.6)		
2.6–3.5	1.175 (0.954–1.447)	0.130
> 3.5	1.223 (0.992–1.508)	0.060
Grade (Reference: Well differentiated)		
Moderately differentiated	1.823 (1.336–2.488)	< 0.001
Poorly differentiated	2.231 (1.574–3.162)	< 0.001
Undifferentiated	2.284 (0.838–6.222)	0.110
Unknown	1.980 (1.445–2.714)	< 0.001
Radiotherapy (Reference: None/Unknown)		
Yes	1.089 (0.915–1.297)	0.340
Chemotherapy (Reference: None/Unknown)		
Yes	0.570 (0.481–0.676)	< 0.001
Surgery (Reference: None/Unknown)		
Yes	0.441 (0.348–0.559)	< 0.001
AJCC T status (Reference: T1)		
T2	1.607 (1.028–2.513)	0.037
T3	1.888 (1.221–2.919)	0.004
T4	1.665 (1.050–2.639)	0.030
Unknown	2.607 (1.579–4.303)	< 0.001
AJCC N status (Reference: N0)		
N1	1.235 (1.065–1.433)	0.005
Unknown	1.383 (0.944–2.026)	0.096
AJCC M status (Reference: M0)		
M1	1.866 (1.568–2.221)	< 0.001
Primary site (Reference: Head of pancreas)		
Body of pancreas	0.861 (0.700–1.059)	0.160
Tail of pancreas	0.903 (0.720–1.133)	0.380
Pancreatic duct	1.438 (0.986–2.098)	0.059
Other specified parts of pancreas	0.958 (0.741–1.238)	0.740
Overlapping lesion of pancreas	1.069 (0.812–1.407)	0.640
Pancreas, NOS	0.864 (0.583–1.280)	0.470
Histological subtype (Reference: Adenocarcinoma)		
Infiltrating duct carcinoma	1.288 (1.061–1.564)	0.011
Neuroendocrine carcinoma	0.195 (0.130–0.292)	< 0.001
Other	0.640 (0.476–0.860)	0.003
Prior thyroid cancer history (Reference: without)		
With	0.722 (0.542–0.961)	0.026

*CSS* cancer-specific survival, *AJCC* American Joint Commission on Cancer(7th), *SEER* the surveillance, epidemiology, and end results, *HR* hazard ratio, *CI* confidence interval.

### Association between the history of thyroid cancer and PC-specific mortality in different gender PC patients

**Figure 4:**
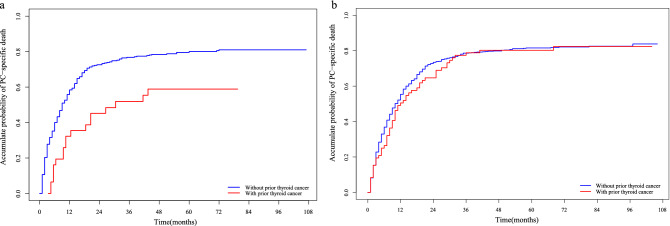
Cumulative incidence of PC-specific death between female and male PC patients with and without prior thyroid cancer. (**a**) Males. (**b**) Females.* PC* pancreatic cancer.

For male PC patients, a history of thyroid cancer could reduce PC-specific mortality by 0.494 times (HR = 0.506, 95% CI 0.281–0.912, *p* = 0.023). On the other hand, for women PC patients, the history of thyroid cancer had no significant effect on PC-specific mortality (HR = 0.883, 95% CI 0.651–1.199, *p* = 0.430). The cumulative PC-specific mortality between PC patients with and without a history of thyroid cancer in males and females are presented in Fig. 4a and b, respectively. The subgroup analysis showed no statistically significant effect of prior thyroid cancer on PC-specific mortality in males and females in different subgroups of PC patients (Supplementary Table 5).

### Association between the history of thyroid cancer and PC-specific mortality in PC patients with different histological subtypes

The PC-specific cumulative mortality significantly varied among PC patients with different histological subtypes, and it was the highest in PC patients with the adenocarcinoma subtype (Supplementary Fig. 2). Therefore, we analyzed the effects of the history of thyroid cancer on PC-specific mortality in PC patients with different histological subtypes. Because the number of PC patients with a history of thyroid cancer in the pancreatic neuroendocrine tumor histological subtype was small, the difference in PC-specific mortality between patients with and without a history of thyroid cancer could not be calculated. For the other three histological subtypes, the history of thyroid cancer had no significant effect on PC-specific mortality (Table 4). The PC-specific mortality between PC patients with and without a history of thyroid cancer in four tumor histological subtypes is shown in Supplementary Fig. 3. Interestingly, the PC-specific mortality in PC patients with a history of thyroid cancer was higher than in those without a history of thyroid cancer in infiltrating duct carcinoma histological subtype.

**Table 4:** Effects of prior thyroid cancer on survival of PC patients based on gender grouping and tumor histological subtype grouping.

Subgroup	Variable	HR (95% CI))	P value
Sex	Female	0.883 (0.651–1.199)	0.430
	Male	0.506 (0.281–0.912)	0.023
Histological subtype	Adenocarcinoma	0.776 (0.561–1.075)	0.130
	Infiltrating duct carcinoma	1.413 (0.755–2.645)	0.280
	Neuroendocrine carcinoma	–	–
	Other	0.239 (0.050–1.133)	0.071

*HR* hazard ratio, *CI* confidence interval.

## Discussion

Cancer survivors have greatly increased in the past few decades thanks to breakthroughs in early diagnosis and treatment, projected to reach 22.1 million in the United States by 2030^16^. Long-term cancer survivors face various consequences of cancer and treatment, including an increased likelihood of a diagnosis of a second primary tumor^17^. The thyroid hormone plays an important role in digestive system tumors^18^. However, the role of a history of thyroid cancer in PC patients’ prognosis remains unclear. Thus, we carried out a SEER population-based study to investigate the impact of prior thyroid cancer on PC. We analyzed PC patients’ OS and CSS and found that age, race, grade, chemotherapy, surgery, AJCC TNM status, and histological subtype were independent predictors of OS and CSS in PC patients. A history of thyroid cancer could reduce PC-specific mortality in PC patients. In the subgroup analysis, the history of thyroid cancer could reduce the PC-specific mortality in male PC patients but not in female ones. The prior thyroid cancer had no significant effect on PC-specific mortality in PC patients with various histological subtypes.

The thyroid hormone can regulate the growth and homeostasis of the digestive tract by binding to hormone receptors^18^. Previous studies have also found that hyperthyroidism can increase the risk of PC^19^. The main treatment option for thyroid cancer is surgery, after which patients would develop hypothyroidism; thus, thyroid hormone replacement therapy is required^20^. One study has found that PC patients with hypothyroidism who took exogenous thyroid hormones had more malignant PC biological behavior and worse prognosis^21^. Moreover, cell line experiments have shown that exogenous thyroid hormones can increase the malignant ability of PC cells^21^. This result suggested that thyroid hormones might be detrimental to survival in PC patients. Thus, we hypothesized that the relatively low thyroid hormone levels in PC patients with prior thyroid cancer might benefit their prognosis. Cancer survivors will strengthen primary tumor monitoring, leading to earlier detection of a second primary tumor^22^. Additionally, cancer survivors might make lifestyle changes after diagnosis or treatment to have a healthier lifestyle^23^. A healthy lifestyle can reduce the risk of recurrence, secondary primary tumors, and chronic cardiovascular disease in these cancer survivors^24,25^.

Herein, the median OS of PC patients with prior thyroid cancer was 12 months, longer than the median OS of 9 months in patients without prior thyroid cancer. Although there was no statistical difference in median OS between the two groups, large-sample prospective studies are still needed to investigate the role of prior thyroid cancer in PC prognosis. A previous study has shown that prior thyroid cancer can reduce the incidence of dying from liver cancer only in female patients^10^. Interestingly, we found reduced PC-specific mortality only in male PC patients with prior thyroid cancer. Thyroid cancer is more likely to occur in females, but male patients have a higher malignant grade and worse prognosis^26^. Older age and later diagnosis of male thyroid cancer patients might be associated with poor prognosis^27^. Estrogen can also enhance the proliferation of thyroid cancer cells^28,29^. Thus, we speculated that differences in estrogen levels between male and female patients might lead to differences in the role of prior thyroid cancer in PC patients.

This was a large population-based retrospective study, and the PSM method was used to reduce confounding factors between groups to increase comparability. The prognosis of thyroid cancer is very different from PC, and we studied the role of prior thyroid cancer in PC prognosis for the first time. However, our current study also has some limitations. First, patients were mostly white and black. Different countries have different races, and disease susceptibility and treatment vary in different countries. Second, the SEER database lacks patient-specific treatment regimens, such as unknown chemotherapy drugs and cycles. Third, this retrospective study needs further verification in prospective research.

## Conclusion

Prior thyroid cancer did not affect the OS of PC patients. However, it reduced PC-specific mortality in male PC patients. The PC-specific cumulative mortality was the highest in PC patients with the adenocarcinoma subtype. These results indicated that attention should be paid to the tumor history in pancreatic cancer treatment, especially the history of thyroid cancer.

## Supplementary Information

Supplementary Information.

## Data Availability

All datasets used in this study can be downloaded from the SEER database (https://seer.cancer.gov/) or obtained from the corresponding author.
