# Supplementary material for: Value of thyroid cancer history in the prognosis of pancreatic cancer: a SEER population-based study
Source: Sci Rep. 2023 Apr 8;13:5771. doi: 10.1038/s41598-023-32635-z (PMC10082804; doi:10.1038/s41598-023-32635-z)
Supplement: Supplementary file 1 — Supplementary Information. [file 41598_2023_32635_MOESM1_ESM.pdf]

Characteristics	Total (n=33431)	Without prior thyroid cancer (n=33328)	With prior thyroid cancer (n=103)	Statistics	P value
**Age at diagnosis, n (%)**				χ2=0.698	0.705
<68	16258(48.6)	16208(48.6)	50(48.5)		
68-79	11301(33.8)	11269(33.8)	32(31.1)		
>79	5872(17.6)	5851(17.6)	21(20.4)		
**Sex, n (%)**				χ2=18.269	<0.001
Female	16342(48.9)	16270(48.8)	72(69.9)		
Male	17089(51.1)	17058(51.2)	31(30.1)		
**Race, n (%)**				χ2=5.593	0.061
Black	4613(13.8)	4603(13.8)	10(9.7)		
White	26188(78.3)	26109(78.3)	79(76.7)		
Other	2630(7.9)	2616(7.8)	14(13.6)		
**Tumor size, n (%)**				χ2=8.427	0.015
<2.6	7391(22.1)	7361(22.1)	30(29.1)		
2.6-3.5	8889(26.6)	887426.6)	15(14.6)		
>3.5	17151(51.3)	17093(51.3)	58(56.3)		
**Grade, n (%)**				Fisher	0.345
Well differentiated	2810(8.4)	2796(8.4)	14(13.6)		
Moderately differentiated	5462(16.3)	5445(16.3)	17(16.5)		
Poorly differentiated	4693(14.0)	4677(14.0)	16(15.5)		
Undifferentiated	286(0.9)	286(0.9)	0(0)		
Unknown	20180(60.4)	20124(60.4)	5654.4)		
**Radiotherapy, n (%)**				χ2=1.086	0.297
None / Unknown	27293(81.6)	27213(81.7)	80(77.7)		
Yes	6138(18.4)	6115(18.3)	23(22.3)		
**Chemotherapy, n (%)**				χ2=0.062	0.804
None / Unknown	13233(39.6)	13191(39.6)	42(40.8)		
Yes	20198(60.4)	20137(60.4)	61(59.2)		
**Surgery, n (%)**				χ2=2.243	0.134
None / Unknown	24268(72.6)	24200(72.6)	68(66.0)		
Yes	9163(27.4)	9128(27.4)	35(34.0)		
**AJCC T status, n (%)**				χ2=3.112	0.539
T1	2202(6.6)	2193(6.6)	9(8.7)		
T2	8032(24.0)	8004(24.0)	28(27.2)		
T3	14410(43.1)	14364(43.1)	46(44.7)		
T4	6372(19.1)	6357(19.1)	15(14.6)		
Unknown	2415(7.2)	2410(7.2)	5(4.9)		
**AJCC N status, n (%)**				χ2=3.347	0.188
N0	18344(54.9)	18280(54.8)	64(62.1)		
N1	12473(37.3)	12438(37.3)	35(34.0)		
Unknown	2614(7.8)	2610(7.8)	4(3.9)		

**Characteristics**	**Total (n=33431)**	**Without prior thyroid cancer (n=33328)**	**With prior thyroid cancer (n=103)**	**Statistics**	**P value**
**AJCC M status, n (%)**				χ2=1.236	0.266
M0	18961(56.7)	18897(56.7)	64(62.1)		
M1	14470(43.3)	14431(43.3)	39(37.9)		
**Primary Site, n (%)**				Fisher	0.028
Head of pancreas	17768(53.1)	17712(53.1)	56(54.4)		
Body of pancreas	5002(15.0)	4996(15.0)	6(5.8)		
Tail of pancreas	5357(16.0)	5333(16.0)	24(23.3)		
Pancreatic duct	99(0.3)	98(0.3)	1(1.0)		
Islets of Langerhans	21(0.1)	219(0.1)	0		
Other specified parts of pancreas	665(2.0)	665(2.0)	0		
Overlapping lesion of pancreas	2531(7.6)	2522(7.6)	9(8.7)		
Pancreas, NOS	1988(5.9)	1981(5.9)	7(6.8)		
**Histological subtype, n (%)**				χ2=1.290	0.734
Adenocarcinoma	23618(70.6)	23550(70.7))	68(66.0)		
Infiltrating duct carcinoma	4239(12.7)	4225(12.7)	14(13.6)		
Neuroendocrine carcinoma	1710(5.1)	1704(5.1)	6(5.8)		
Other	3864(11.6)	3849(11.5)	15(14.6)		
**Vital status, n (%)**				χ2=2.139	0.144
Alive	3963(11.9)	3946(11.8)	17(16.5)		
Dead	29468(88.1)	29382(88.2)	86(83.5)		
**SEER cause death classification, n (%)**				χ2=5.696	0.058
Alive	3963(11.9)	3946(11.8)	17(16.5)		
Death due to other causes	1912(5.7)	1902(5.7)	10(9.7)		
Death due to pancreatic cancer	27556(82.4)	27480(82.5)	76(73.8）		
Survival month, M (Q1, Q3)	9.0(3.0,21.0)	9.0 (3.0,21.0)	11.0(5.0,29.0)	Z=-2.001	0.045

Supplementary Table 1. Patients' characteristics before propensity score matching. AJCC, American Joint Committee on Cancer(7th). SEER, the Surveillance, Epidemiology, and End Results.

**Characteristics**	**Total (n=103)**
**Age at diagnosis, mean ± SD**	62.5±13.8
**Sex, n (%)**	
Female	72(69.9)
Male	31(30.1)
**Race, n (%)**	
Black	10(9.7)
White	79(76.7)
Other	14(13.6)
**Tumor size, n (%)**	
<3.0	60(58.2)
≥3	21(20.4)
Unknown	22(21.4)
**Grade, n (%)**	
Well differentiated	11(10.7)
Moderately differentiated	6(5.8)
Undifferentiated	1(1.0)
Unknown	85(82.5)
**Radiotherapy, n (%)**	
None / Unknown	57(55.3)
Yes	46(44.7)
**Chemotherapy, n (%)**	
None / Unknown	103(100)
**Surgery, n (%)**	
None / Unknown	5(4.9)
Yes	98(95.1)
**AJCC T status, n (%)**	
T1	48(46.6)
T2	10(9.7)
T3	17(16.5)
T4	7(6.8)
Unknown	21(20.3)
**AJCC N status, n (%)**	
N0	71(68.9)
N1	12(11.6)
Unknown	20(19.4)
**AJCC M status, n (%)**	
M0	82(79.6)
M1	1(1.0)
Unknown	20(19.4)
**Survival month, M (Q1, Q3)**	75.0(44.0,127.0)
**Vital status, n (%)**	
Alive	17(16.5)
Dead	86(83.5)

Characteristics	Total (n=103)
**SEER cause death classification, n (%)**	
Alive or dead of other cause	97(94.2)
Dead (attributable to this cancer)	6(5.8)
**Histological subtype, n (%)**	
Papillary Subtype of the thyroid	52(50.5)
Follicular Subtype of the thyroid	4(3.9)
Mixed medullary and follicular cell carcinoma	1(1.0)
Other carcinomas of thyroid gland	44(42.7)
Unknown	2(1.9)

Supplementary Table 2. Characteristics of 103 thyroid cancer patients. AJCC, American Joint Committee on Cancer(6th). SEER, the Surveillance, Epidemiology, and End Results.

PC Patients	Without prior thyroid cancer history (The median OS)	With prior thyroid cancer history (The median OS)	P value
Subgroup 1	9	10	0.420
Subgroup 2	—	—	0.550
Subgroup 3	12	—	0.008

Supplementary Table 3. KM survival analysis of different subgroups. — indicates that the median OS is not reached. OS, overall survival.

Subgroup	Univariate analysis (Reference: without Prior thyroid cancer history)		Multivariate analysis (Reference: without Prior thyroid cancer history)	
	HR (95% CI)	P value	HR (95% CI)	P value
Subgroup 1	0.924(0.730-1.170)	0.512	0.953(0.745-1.218)	0.702
Subgroup 2	1.808(0.545-5.994)	0.332	0.471(0.071-3.144)	0.437
Subgroup 3	0.468(0.216-1.014)	0.054	0.273(0.109-0.684)	0.005

Supplementary Table 4. Univariate and multivariate Cox survival analysis of OS in different subgroups of PC patients.

Subgroup	Gender	HR (95% CI))	P value
Subgroup 1	Female	0.947(0.682 -1.316)	0.750
	Male	0.646(0.347-1.201)	0.170
Subgroup 2	Female	——	——
	Male	——	——
Subgroup 3	Female	0.460(0.100-2.120)	0.320
	Male	——	——

Supplementary Table 5. Effects of prior thyroid cancer on PC-specific mortality in males and females in different subgroups of PC patients. HR, hazard ratio. CI, confidence interval.

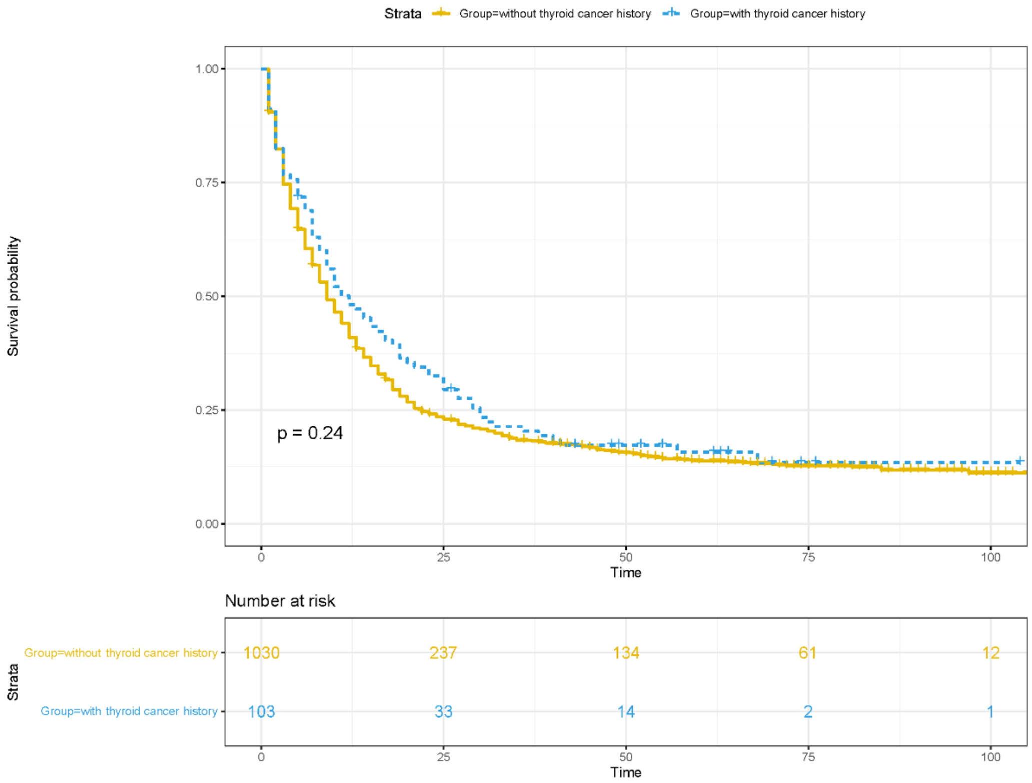

Supplementary Figure 1. The KM survival curves between PC patients with and without prior thyroid cancer.

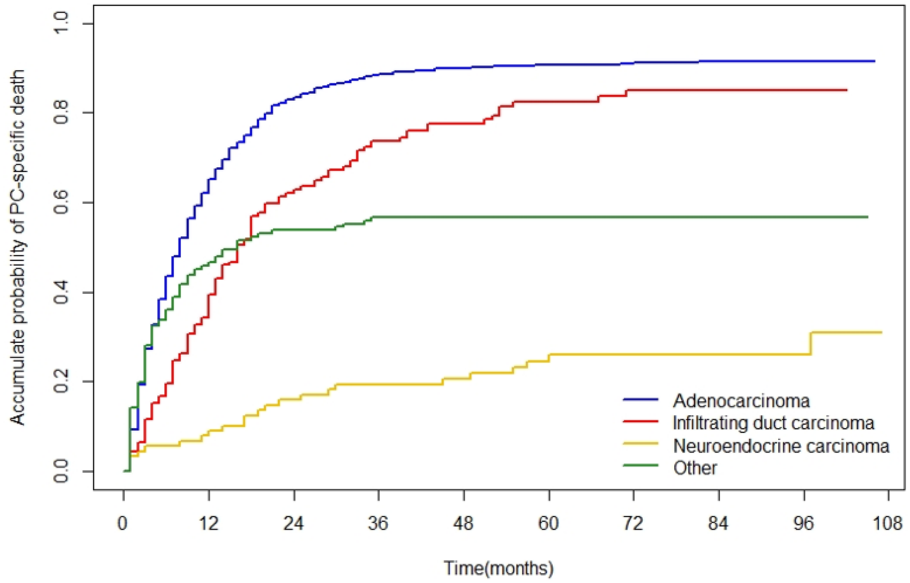

Supplementary Figure 2. The cumulative incidence of PC-specific death in four histological subtypes.

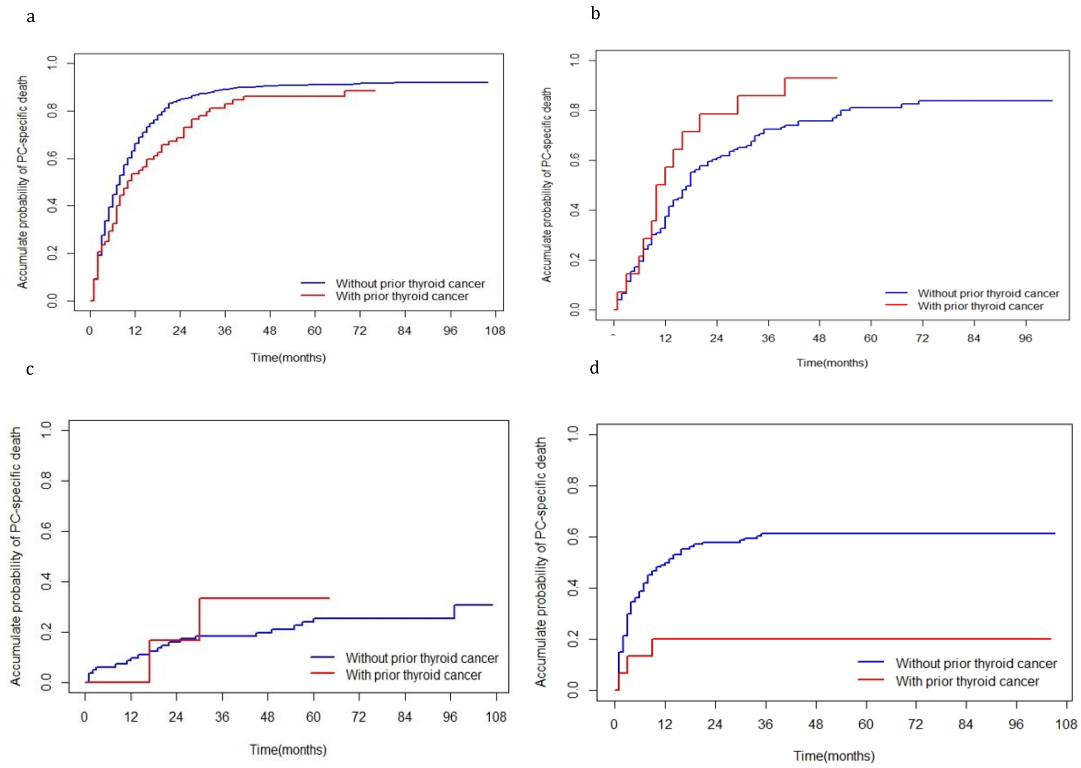

Supplementary Figure 3. The cumulative incidence of PC-specific death between PC patients with and without prior thyroid cancer in all histological subtypes. (a)The cumulative incidence of PC-specific death between PC patients with and without prior thyroid cancer in adenocarcinoma.(b) The cumulative incidence of PC-specific death between PC patients with and without prior thyroid cancer in infiltrating duct carcinoma.(c) The cumulative incidence of PC-specific death between PC patients with and without prior thyroid cancer in neuroendocrine carcinoma.(d) The cumulative incidence of PC-specific death between PC patients with and without prior thyroid cancer in other histological subtypes.
